# Improving resolution of panoramic radiographs: super-resolution concept

**DOI:** 10.1093/dmfr/twae009

**Published:** 2024-03-14

**Authors:** Mahmut Emin Çelik, Mahsa Mikaeili, Berrin Çelik

**Affiliations:** Electrical Electronics Engineering Department, Faculty of Engineering, Gazi University, Ankara, Eti Mh. Yükselis sk. No:5, 06570, Turkey; Biomedical Calibration and Research Center (BIYOKAM), Gazi University Hospital, Gazi University, Ankara, 06560, Turkey; Biomedical Calibration and Research Center (BIYOKAM), Gazi University Hospital, Gazi University, Ankara, 06560, Turkey; Oral and Maxillofacial Radiology Department, Faculty of Dentistry, Ankara Yıldırım Beyazıt University, Ankara, Yayla Mahallesi Yozgat Bulvarı, 1487. Cadde No:55, 06010, Turkey

**Keywords:** radiographic magnification, deep learning, dentistry, panoramic, X-ray, artificial intelligence

## Abstract

**Objectives:**

Dental imaging plays a key role in the diagnosis and treatment of dental conditions, yet limitations regarding the quality and resolution of dental radiographs sometimes hinder precise analysis. Super-resolution with deep learning refers to a set of techniques used to enhance the resolution of images beyond their original size or quality using deep neural networks instead of traditional image interpolation methods which often result in blurred or pixelated images when attempting to increase resolution. Leveraging advancements in technology, this study aims to enhance the resolution of dental panoramic radiographs, thereby enabling more accurate diagnoses and treatment planning.

**Methods:**

About 1714 panoramic radiographs from 3 different open datasets are used for training (*n* = 1364) and testing (*n* = 350). The state of the art 4 different models is explored, namely Super-Resolution Convolutional Neural Network (SRCNN), Efficient Sub-Pixel Convolutional Neural Network, Super-Resolution Generative Adversarial Network, and Autoencoder. Performances in reconstructing high-resolution dental images from low-resolution inputs with different scales (*s* = 2, 4, 8) are evaluated by 2 well-accepted metrics Structural Similarity Index (SSIM) and Peak Signal-to-Noise Ratio (PSNR).

**Results:**

SSIM spans between 0.82 and 0.98 while PSNR are between 28.7 and 40.2 among all scales and models. SRCNN provides the best performance. Additionally, it is observed that performance decreased when images are scaled with higher values.

**Conclusion:**

The findings highlight the potential of super-resolution concepts to significantly improve the quality and detail of dental panoramic radiographs, thereby contributing to enhanced interpretability.

## Introduction

The resolution of imaging methods in medicine including X-ray imaging, MRI, and ultrasound imaging is paramount as it facilitates early detection, enabling clinicians to intervene promptly, thus preventing the progression of diseases and reducing potential complications. However, they are prone to have low-resolution as a results of different factors and situations. In dentistry, low-resolution dental radiographs can present several disadvantages that might impact diagnostic accuracy and quality of patient care.^1^ Reduced image clarity lack detail and clarity in visualization, making it harder for dentists to identify small lesions, cracks, or other subtle abnormalities in the teeth or surrounding structures.^2^^,^^3^ It brings limited diagnostic accuracy, inability to detect cases and eventually increased risk of errors.

Moreover, dental radiographs tend to have a higher level of noise, artefacts, blur, and aliasing effects which in turn reduce the images quality and consequently accurate interpretation.^1^^,^^4^ There are 3 main factors causing low resolution (LR) dental radiographs, firstly device-specific conditions in terms of the limitation of acquisition tools, detector geometry, sensitivity, reconstruction technics, secondly patient-related issues like movement, lastly technician-related issues like incorrect positioning of the imaging equipment, and inadequate exposure settings.^2–4^ Considering the complexity of the oral cavity and the presence of hard and soft tissues in a small volume, it can make it difficult to obtain high quality images.

To address these issues and enhance interpretation of dental radiographic images, zooming tools which generally rely on conventional interpolation methods are used up to a limit.^5^^,^^6^ Magnifying images beyond this limit point make it potentially harder to define the boundaries of anatomical structures and lesions and decrease diagnostic accuracy.

In recent years, artificial intelligence, especially deep neural networks present satisfactory results in dental image analysis.^7–13^ As an emerging research field, super-resolution is a technique used in image processing and computer vision to enhance the resolution and quality of an image beyond its original resolution. Deep learning concept has been extensively employed to achieve super-resolution. The goal of super-resolution is to generate a high-resolution image from one or more low-resolution input images. This can be particularly useful in various applications, including medical imaging. It refers the process of obtaining an image with better details than the current sample by increasing the number of pixels per unit area. Applying deep learning models for super-resolution of dental radiographs may offer to provide benefits. It can assist dentists and oral health professionals in better identifying and analysing dental pathologies, caries, fractures, bone density, root canal anatomy, and other oral health issues thanks to enhanced resolution and quality of dental images obtained from different modalities such as X-rays.^14^ Moreover, in remote or underserved areas where access to specialized dental care might be limited, telemedicine and tele dentistry with super-resolution can play a crucial role. Super-resolution can assist in getting higher-quality images for remote consultation and diagnosis. Additionally, for educational purposes, clearer images can aid in teaching dental students and professionals by providing better visual materials for learning. Previously, Moran et al applied CNN to enhance the quality and resolution of periapical radiographic images.^4^ Wang et al used deep learning model to provide clearer details and textures from Dental CT and facilitate effective diagnosis.^15^ Li et al investigated a super-resolution model using panoramic radiographs for predicting mandibular third molar extraction difficulty.^16^ Hatvaniy et al adopted single image super-resolution (SISR) technique by employing Tucker decomposition for denoising and deconvolution of 3D dental CT images.^17^ Hatvani et al used super-resolution concept to enhance spatial resolution of 2 dimensional Cone Beam Computed Tomography images.^3^ Rahimi et al compared super-resolution performances of deep learning models and convolutional techniques using panoramic radiographs.^18^ Hwang et al addressed the storage space and cost issues associated with storing large-capacity CBCT data in dentistry.^19^

This work aims to apply super-resolution concept with deep learning and Generative Adversarial Network (GAN) to enhance the resolution of dental radiographs. Panoramic radiographs from 3 different open datasets are used for training and testing. The state-of-the-art 4 different models is applied, namely Super-Resolution Convolutional Neural Network (SRCNN), Efficient Sub-Pixel Convolutional Neural Network (ESPCNN), Super-Resolution Generative Adversarial Network (SRGAN), and Autoencoder. Performances are evaluated by 2 well-accepted metrics Structural Similarity Index (SSIM) and Peak Signal-to-Noise Ratio (PSNR). Findings are promising and indicate that super-resolution concept can be useful tool for improving dental image analysis.

## Methods

### Data preparation and scaling

Large balanced dataset is of great importance for deep learning-based research. Panoramic radiographs from 3 different publicly available open datasets are used. They are Tufts Dental Database, Panoramic radiography database, and Panoramic Dental X-rays with Segmented Mandibles.^20–22^ They included 1000, 598, and 116 panoramic radiographs respectively. Images were split into training and test sets with the ratio of 80% and 20%, which corresponds to 1364 images for training and 350 images for test. Information regarding the selection of the sample, patient population, and inclusion and exclusion criteria were briefly presented in Table 1:

**Table 1.** Criteria applied for the used 3 open-source dental datasets.

Dataset	Inclusion criteria	Exclusion criteria	Device
Panoramic Dental Xray Dataset ^19^	Panoramic images taken for diagnosis and treatment purposes	Records with implants Patients below 20 years of age Low-quality x-rays, which were blurred or malposed	Soredex CranexD digital panoramic x-ray unit
Tufts Dental Database ^20^	Optimum diagnostic quality of the image with minimal or no technical errors in the image	Not reported	OP100 Orthopantomograph (Instrumentarium Imaging/Kavo Kerr) Planmeca Promax 2D (Henry Schein)
Panoramic radiography database^21^	Complete dentition	Images without restorations and without radiographically detectable pathologies	I-Max touch, Owandy Radiology, France

**Figure 1.**
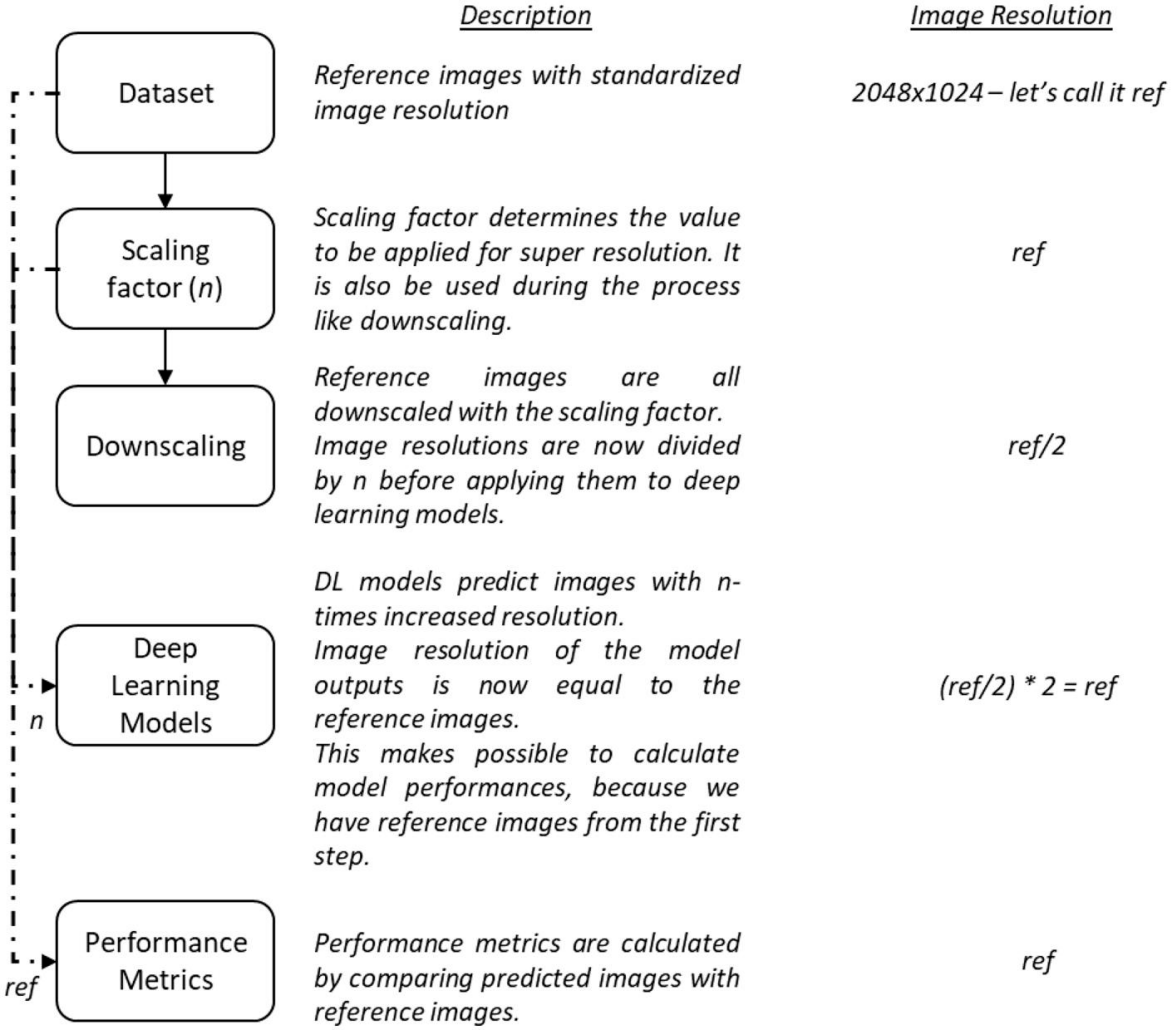
Super-resolution using deep learning step by step explanation in terms of image resolution, explanations for the term scaling factor.

Since the sizes of the images in different data sets were different from each other, all images were normalized to be the same size 2048 × 1024 pixels, which were assumed as reference. To evaluate the networks performance in increasing images’ resolution, reference images were downscaled by scaling ratios of 2, 4, and 8. By doing so, models were trained with images scaled by 2, 4, and 8, which make possible to evaluate models’ performances in terms of super-resolution. Figure 1 shows procedures regarding images from beginning to the end step by step with explanations and exact image resolutions as different columns at the respective steps.

During training the number of epochs was settled to 50. The experimentation was conducted using an Intel eighth generation i7 processor unit with 4 GHz and 16 GB of RAM, along with an NVIDIA GeForce GTX 1050 graphics card.

### Deep convolutional neural networks for super-resolution

SR defines it as a process of acquiring high resolution (HR) images from LR ones. It attempts to produce better details while enhancing the actual resolution.^23^^,^^24^ SR tasks assess as an ill-posed problem based on eqn (1).^24^

(1) $$L\left(i,j\right)=D\left(B\left(W\left(H\left(i,j\right)\right)\right)\right)+\eta (i,j)$$

Where in eqn (1), $L\left(i,j\right)$ and H(i,j) indicates LR and HR images, respectively. Also W is warping function, B is blurring function, and D indicates downscaling. The process of generating images with higher resolution can be divided into 2 subcategories. One of them is relied on single images super-resolution and the other one based on video frames or multiple images. Single image SR is well suited for the aim of enhancing resolution of radiographic images. Moreover, super-resolution could be classified as traditional or conventional ones and deep learning-based methods. Conventional methods rely on interpolation methods such as bilinear, bicubic, lanczos as well as reconstruction-based methods which are prone to degrading by increasing scaling factor.^25^

**Figure 2.**
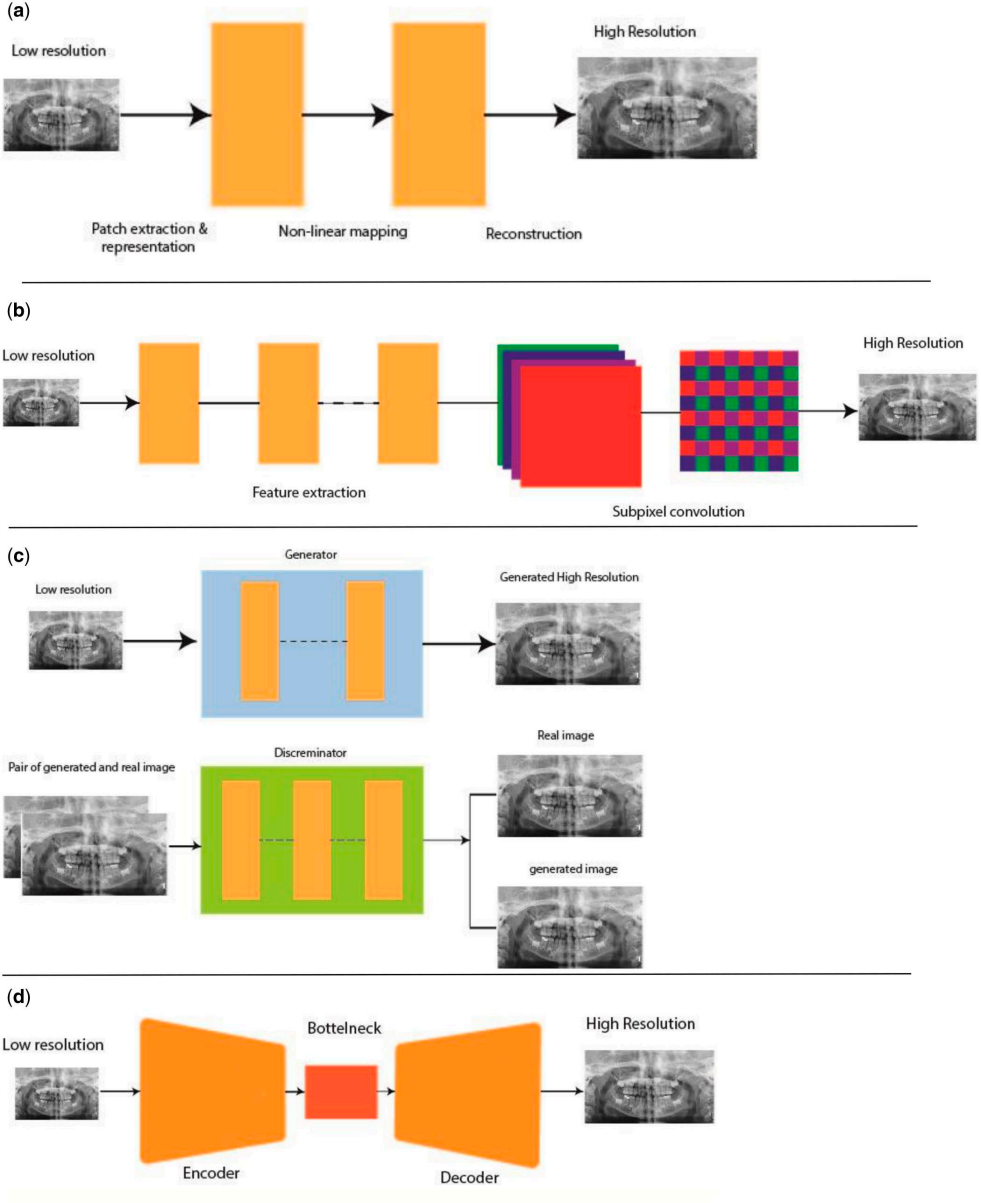
Schematic diagrams of the models implemented for SRCNN (A), ESPCN (B), SRGAN (C), and Autoencoder (D).

The first network is SRCNN.^26^ This network is capable of learning directly in an end-to-end manner by mapping between low- and high-resolution images. It includes a moderate number of filters and layers which allows for fast and practical usage. It consists of 3 inputs, hidden and output layers respectively. The input layer takes LR images and the output layer produces high-resolution images. The hidden layers are the core of the structure and could encompass multiple convolutional layers and the best trade off acquired with 3 convolutional layers. The first hidden layer extracts feature from the input image. The second convolutional layer maps the extracted features to a higher dimensional space and ultimately third convolutional layer maps higher dimensional features to the output. Each convolutional layer followed by ReLU activation function Figure 2.

The second network is ESPCN which performs real-time SISR.^27^ This network takes LR images as an input and then passes them through 2 convolutional layers to extract feature maps. Subsequently, output of these layers passes through a subpixel convolutional layer aiming to accumulate the feature maps from LR space and construct HR images in a single step. It also employs one upscaling feature map for creating HR images directly from LR ones.

The third network is SRGAN.^28^ It includes 2 deep neural network structures namely generator and discriminator networks. The generator networks take LR images and attempt to produce HR ones in the output. The discriminator networks take the HR images which are produced by generator as an input and then classified it as real HR or fake images. The generator network is trained to produce super-resolved images that are classified as real by the discriminator network, while the discriminator network is trained to distinguish between real high-resolution images and super-resolved images produced by the generator network.^28^

Lastly, Autoencoder structure is a type of generative model which consists of encoder and decoder parts. The former encodes the input data which represents it in many magnitudes order smaller than input images (lower dimensional representation of feature maps) while the latter reconstructs data from encoded forms. Autoencoder is capable of capturing most important parts of input images.

### Evaluation metrics

There are 2 evaluation metrics applied in the present work, namely SSIM and PSNR. SSIM measures image quality and determines how similar the predicted image with HR is to the reference image.^29^^,^^30^ The SSIM index calculation relies on 3 main aspects as luminance, contrast and structural similarities as given in eqn (2).

(2) $$\mathrm{SSIM}=\;\frac{\left(2\mu_{x}\mu_{y}+c_{1})(2\sigma_{xy}+c_{2}\right)}{\left(\mu_{x}^{2}+\mu_{y}^{2}+c_{1})(\sigma_{x}^{2}+\sigma_{y}^{2}+c_{2}\right)}$$

In which $\mu_{x}$ and $\mu_{y}$ are averages for x and y windows. Also, $\sigma_{x}^{2}$ and $\sigma_{y}^{2}$ are their related variances. The goal of $c_{1}$ and $c_{2}$ is to keep the denominator from going to zero.

The other evaluation metric, PSNR refers to absolute error in decibels between the reference image and the predicted image with HR. Mathematical representation of the PSNR is given in eqn (3) where MSE indicates mean square error between the acquired and reference images. Higher values refer to high performance, with the maximum value of 50.

(3) $$\mathrm{PSNR}=10\times \log_{10}(\frac{{\left(2^{n}-1\right)}^{2}}{\mathrm{MSE}})$$

## Results

Models are trained and tested with 3 different scaling 2, 4, and 8. Findings were presented in Table 2. SSIM and PSNR were evaluated for 4 deep learning models with 3 different scales. It was seen that increasing scaling decreased performance for all models. For all scales, SSIM were between 0.8228 and 0.9822 while PSNR spans between 28.74 and 40.28. For scale 2, SRCNN and ESPCN yielded significant performance with SSIM of higher than 0.98. The corresponding PSNR was higher than 39.

**Table 2.** Findings of super-resolution with the ratios of 2, 4, and 8 using deep learning.

	Scale: 2			
	SRCNN	ESPCN	SRGAN	Autoencoder
**SSIM**	0.9822	0.9802	0.9658	0.9749
**PSNR**	40.2832	39.7264	36.9737	38.4860
	**Scale: 4**			
**SSIM**	0.9441	0.9422	0.9022	0.9368
**PSNR**	34.8279	34.5374	32.1900	33.8873
	**Scale: 8**			
**SSIM**	0.9058	0.9044	0.8228	0.8992
**PSNR**	31.4242	31.2154	28.7454	30.6329

**Figure 3.**
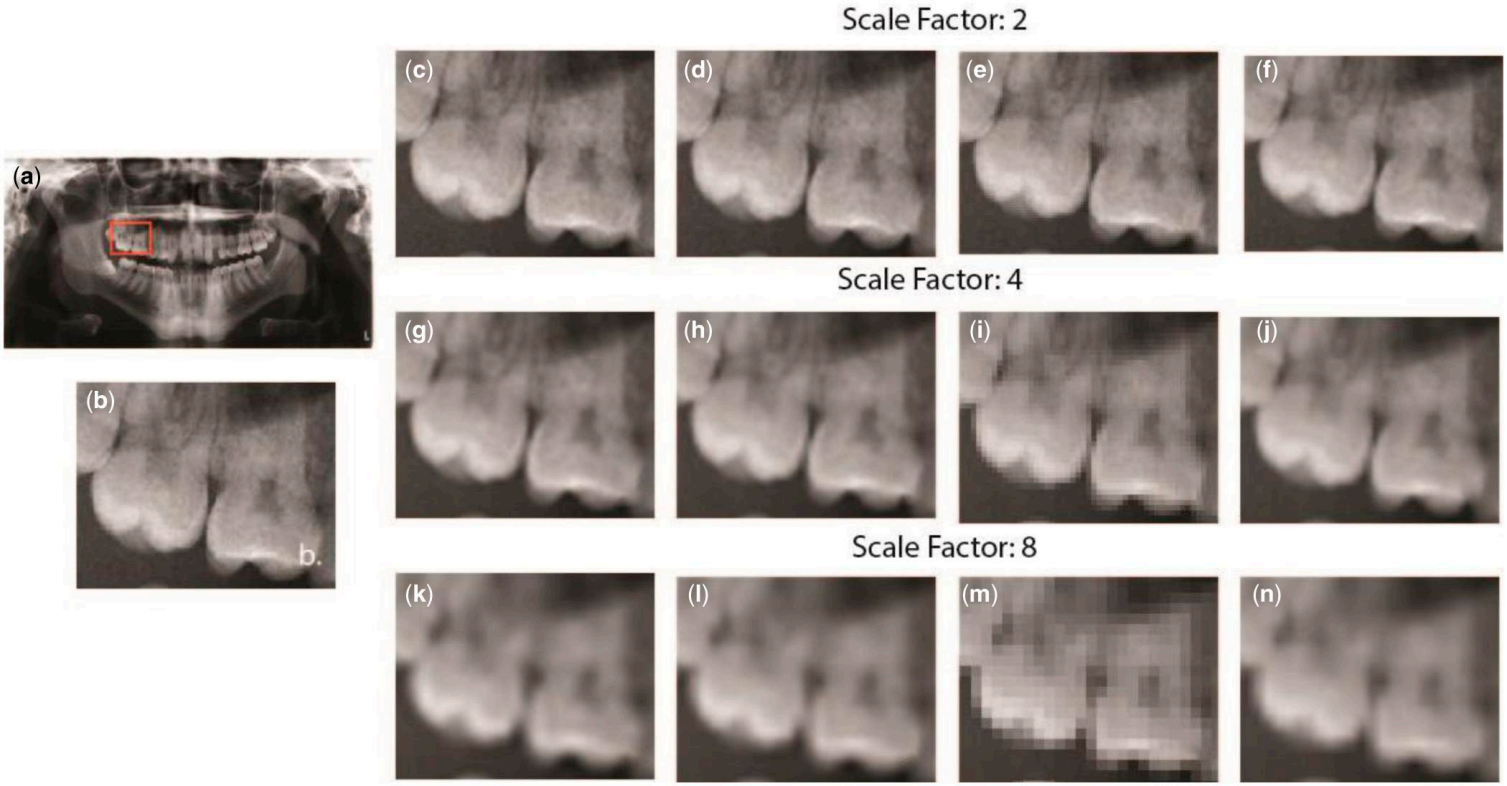
Results from different deep learning models for 3 scale factors. (A) original image, (B) selected region. (C, G, K) acquired results from SRCNN for 3 scaling factor, (D, H, L) acquired results from ESPCN for 3 scaling factor, (E, I, M) acquired results from SRGAN for 3 scaling factor, (F, J, N) acquired results from Autoencoder for 3 scaling factor.

Figure 3 presents a panoramic radiograph as a reference image. For visualization of the results, the reference image was marked with randomly selected region. Together with the original selected region, the models’ outputs for each scaling were also presented. It was again seen that increased scaling leads to blurring.

**Figure 4.**
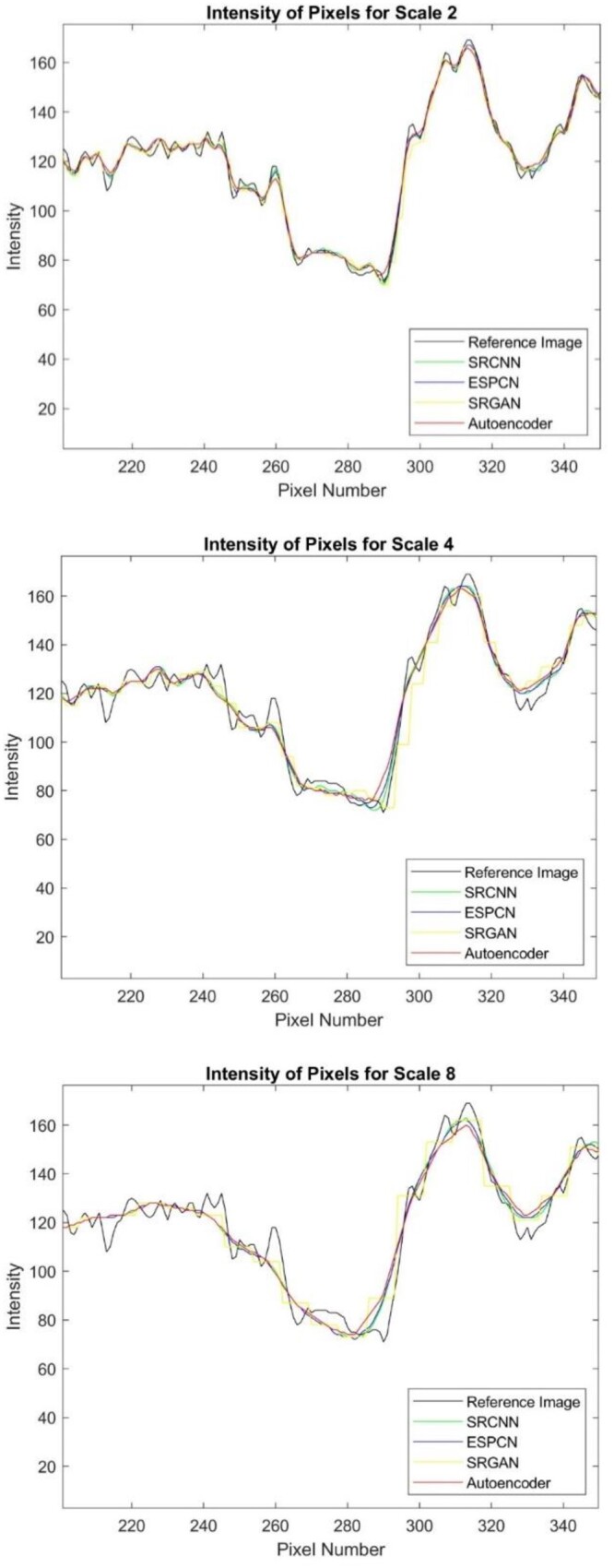
Comparison of images intensity in a specific row for varying scale factor.

Figure 4 compares pixel intensities of selected region with reference image. ESPCN and SRCNN approximated well to the reference image intensity (black line) especially for scaling factors of 2 and 4 while Autoencoder (red line) provided lower performance.

## Discussion

Deep learning methods have rapidly gained prominence across diverse fields in dentistry due to their remarkable capabilities in data analysis, pattern recognition, and image processing.^9^^,^^10^^,^^13^^,^^31^ The combination of these sophisticated algorithms with dental sciences has unveiled new frontiers, offering innovative solutions and augmenting conventional practices in sub-disciplines of dentistry for diagnosis and treatment planning.^8^^,^^11^^,^^12^ Furthermore, deep learning is a promising technology particularly for super-resolution too, facilitating the reconstruction of high-resolution images from lower-resolution inputs. It is a new and emerging trend that deep learning techniques have been increasingly employed in super-resolution tasks in dentistry due to their ability to learn complex patterns and generate images with higher resolution. Eventually, enhanced dental X-ray images with improved visualization can help clinicians to identify many different types of dental conditions.

The present work principally applied Convolutional Neural Networks and GANs to down-scaled images. In the training process, down-scaled images are used instead of reference images for the following reason: when the resolution is increased by a factor of 2, 4, and 8 with deep learning methods, the model outputs can be compared with the reference image to evaluate how accurate the deep learning models are to the reference image with the relevant metrics, which would not be possible otherwise.

There are several previous studies including deep learning for super-resolution in dentistry. They were properly investigated and listed with available information reported to compare with the present work. Table 3 presents comparison with previous works in terms of purpose, type of image, method, and evaluation metric reported. In the previous works, Moran et al used 228 periapical radiographs for SRGAN with and without transfer learning to evaluate super-resolution performance.^4^ The common evaluation metrics, PSNR and SSIM, were reported as 36.99 and 1. Moran et al used periapical radiographs for SR application with deep learning models, SRGAN and SRCNN to assess periodontal bone loss.^1^ Significant visual improvements were noted with PSNR and SSIM of 42.7 and 1, respectively. Wang et al used 2400 dental computed tomography images for deep learning model.^15^ High-resolution images were firstly sampled 4 times by the Bicubic interpolation method. PSNR and SSIM were reported as 36.319 and 0.882. Li et al investigated the effect of super-resolution for predicting mandibular third molar extraction evaluation in panoramic radiographs using 608 panoramic radiographs.^16^ It was reported that SR outperformed the other model for the prediction of mandibular third molar extraction difficulty based on area under the curve (AUC) 0.963 versus 0.821. Hatvaniy et al adopted SISR technique by employing Tucker decomposition for denoising and deconvolution of 3D dental CT images.^17^ Hatvani et al used 7504 2-D CBCT image slices from 17 teeth for training and testing of deep learning models to evaluate resolution enhancement ability.^3^ Results were compared with 2 reconstruction-based SR methods, resulting in the superiority of the CNN models. PSNR was 23.79 and 24.50 for U-Net and Subpixel networks, respectively. Rahimi et al compared super-resolution performances of deep learning models and convolutional techniques using panoramic radiographs.^18^ About 888 panoramic radiographs were used, resulting in SSIM and PSNR of 39.74 ± 0.17 and 0.919 ± 0.003, respectively for the best-performing model. Hwang et al addressed the storage space and cost issues associated with storing large-capacity CBCT data in dentistry.^19^ The network called very deep super-resolution was applied to restore compressed virtual CBCT images using publicly available data. The network enhanced the resolution from low-resolution CBCT images, resulting in superior quality compared to bicubic interpolation.

**Table 3.** Comparison with previous studies on super-resolution.

Author	Purpose	Image type-Size	Applied DNN method	Results-evaluation metrics reported (best model)
Moran et al^4^	Investigate SRGAN and transfer learning for SR	Periapical radiographs	SRGAN	MSE = 14.79 PSNR = 36.99 SSIM = 1.00 MOS = 3.40
Moran et al^1^	Evaluate the impact of different SR methods on the assessment of periodontal bone loss	Periapical radiographs	SRGAN SRCNN	MSE = 0.3.521 PSNR = 42.771 SSIM = 1.00
Wang et al^15^	Super-resolution of dental CT	Dental Ct	SwinIR C-SwinIR	MSE = 16.044 PSNR = 36.319 SSIM = 0.882
Li et al^16^	Prediction of the extraction difficulty for mandibular third molars	Panoramic radiographs	CNNs	AUC = 0.963
Hatvani et al^3^	Resolution enhancement for 2-D CBCT images	CBCT	U-Net Subpixel network	PSNR = 24.50 SSI = 0.8182 IFC = 0.5536 NQM = 8.64
Rahimi et al^18^	Comparison of deep learning models for SR	Panoramic radiographs	SRCNN SRGAN U-Net SwinIr LTE	MSE = 7.47 PSNR = 39.56 SSIM = 0.917
Hwang et al^19^	Exploring super-resolution capability for restoring compressed CBCT images	CBCT	CNN	PSNR: 29.8-40.4 SSIM: 0.9-1
This work	Super-resolution of panoramic dental X-ray	Panoramic radiographs	SRCNN SRGAN ESPCN Autoencoder	PSNR = 40.2832 SSIM = 0.9822

Despite providing the mentioned benefits, these applications come with some challenges and limitations. First, AI-driven applications demand large number of images for training. Second, large balanced multi-centered data are valuable for generalization towards clinical use. Lastly, large amounts of data for AI-driven resolution enhancement methods requires powerful and expensive hardware such as GPU or specialized hardware accelerators.

## Conclusion

This study underscored the transformative potential of employing super-resolution techniques, specifically deep learning-based models, to enhance the resolution and quality of dental panoramic radiographs using a wide range of scaling. The utilization of state-of-the-art deep learning models demonstrated promising outcomes in augmenting the resolution of these radiographs, thereby enabling more accurate diagnoses and treatment planning in dental care.
